# A method for delivering the required neutron fluence in an accelerator-based boron neutron capture therapy system employing a lithium target

**DOI:** 10.1038/s41598-024-62060-9

**Published:** 2024-05-16

**Authors:** Satoshi Nakamura, Mihiro Takemori, Tetsu Nakaichi, Yasunori Shuto, Tairo Kashihara, Kotaro Iijima, Takahito Chiba, Hiroki Nakayama, Yuka Urago, Shuka Nishina, Yuta Kobayashi, Hironori Kishida, Shoji Imamichi, Kana Takahashi, Mitsuko Masutani, Hiroyuki Okamoto, Teiji Nishio, Jun Itami, Hiroshi Igaki

**Affiliations:** 1 Division of Radiation Safety and Quality Assurance, National Cancer Center Hospital, 5-1-1 Tsukiji, Chuo-ku, Tokyo 104-0045 Japan; 2 Division of Boron Neutron Capture Therapy, National Cancer Center Exploratory Oncology Research and Clinical Trial Center, 5-1-1 Tsukiji, Chuo-ku, Tokyo 104-0045 Japan; 3 Medical Physics Laboratory, Division of Health Science, Graduate School of Medicine, Osaka University, 1-7 Yamadaoka, Suita City, Osaka 565-0871 Japan; 4 Department of Radiation Oncology, National Cancer Center Hospital, 5-1-1 Tsukiji, Chuo-ku, Tokyo 104-0045 Japan; 5 Department of Comprehensive Oncology, Nagasaki University Graduate School of Biomedical Sciences, 5-1-1 Tsukiji, Chuo-ku, Tokyo 104-0045 Japan; 6 Department of Radiological Technology, National Cancer Center Hospital, 5-1-1 Tsukiji, Chuo-ku, Tokyo 104-0045 Japan; 7 Department of Radiological Sciences, Graduate School of Human Health Sciences, Tokyo Metropolitan University, 7-2-10 Hitashi-ogu, Arakawa-ku, Tokyo 116-8551 Japan; 8 Department of Radiological Sciences, Komazawa University, 1-23-1 Komazawa, Setagaya-ku, Tokyo 154-8525 Japan; 9 Central Radioisotope Division, National Cancer Center Research Institute, 5-1-1 Tsukiji, Chuo-ku, Tokyo 104-0045 Japan; 10 Department of Molecular and Genomic Biomedicine, Nagasaki University Graduate School of Biomedical Sciences, Sakamoto 1-12-4, Nagasaki, 852-8523 Japan; 11 Radiation Therapy, Shin-Matsudo Central General Hospital, 1-380 Shin-Matsudo, Matsudo City, Chiba 270-0034 Japan

**Keywords:** Boron neutron capture therapy (BNCT), Accelerator-based BNCT, Neutron fluence, Li target, Radiotherapy, Medical research

## Abstract

Accelerator-based boron neutron capture therapy (BNCT) systems employing a solid-state lithium target indicated the reduction of neutron flux over the lifetime of a target, and its reduction could represent the neutron flux model. This study proposes a novel compensatory approach for delivering the required neutron fluence and validates its clinical applicability. The proposed approach relies on the neutron flux model and the cumulative sum of real-time measurements of proton charges. The accuracy of delivering the required neutron fluence for BNCT using the proposed approach was examined in five Li targets. With the proposed approach, the required neutron fluence could be delivered within 3.0%, and within 1.0% in most cases. However, those without using the proposed approach exceeded 3.0% in some cases. The proposed approach can consider the neutron flux reduction adequately and decrease the effect of uncertainty in neutron measurements. Therefore, the proposed approach can improve the accuracy of delivering the required fluence for BNCT even if a neutron flux reduction is expected during treatment and over the lifetime of the Li target. Additionally, by adequately revising the approach, it may apply to other type of BNCT systems employing a Li target, furthering research in this direction.

## Introduction

Many studies have reported favorable clinical outcomes of using boron neutron capture therapy (BNCT) in experimental reactors^1–10^. However, BNCT has not been widely used for cancer treatment because regulations make it difficult to install a nuclear reactor as the neutron source for BNCT in hospitals. Owing to research and developments in BNCT, an accelerator-based neutron source can deliver sufficient neutrons to conduct BNCT and replace nuclear reactors as the source. This can facilitate the clinical implementation of BNCT because the regulations for accelerator-based neutron sources are comparable to those of conventional radiotherapies, such as photon, electron, and particle therapies. Accelerator-based BNCT (AB-BNCT) systems have already been clinically implemented, reporting favorable clinical outcomes^11–19^.

An accelerator-based neutron source generates neutrons via collisions between accelerated particles and target materials. Moreover, several types of the accelerator-based neutron sources have been clinically implemented based primarily on their neutron generation methods^15–19^. One method generates neutrons via the ^9^Be(p, n)^9^B reaction, whereas another utilizes the ^7^Li(p, n)^7^Be reaction. The National Cancer Center Hospital (NCCH) in Tokyo, Japan, installed an AB-BNCT system employing solid-state Li as the target material and the ^7^Li(p, n)^7^Be reaction for generating neutrons^20,21^. However, AB-BNCT systems have challenges with neutron generation^20^. One, in particular, is the degradation of the Li target, which reduces the neutron flux per unit of proton current depending on the total number of protons delivered to the target material^20–22^.

In conventional photon therapy, the photons are monitored by a real-time measurement device in a medical linear accelerator (LINAC); subsequently, the beam on/off control utilizes these measurements to deliver a prescribed dose to a patient. Hence, to follow clinical protocols, a comparable method to deliver the prescribed dose to a patient is required for AB-BNCT systems to control the neutron beam^23^.

Previous studies show that in AB-BNCT systems employing solid-state Li targets, the neutron flux per unit proton current decreases over the lifetime of the target. This reduction is also expected during treatment^21,22^. A previous study indicated that the reduction could be represented by a neutron flux model, which was a function of the total number of protons delivered to the target material^22^. The neutron flux model was established by the neutron yield of the ^7^Li(p, n) reaction by considering the incident proton energy and each thickness of the Li target. To achieve a sufficient number of neutrons in an AB-BNCT system using a Li target, a large quantity of the ^7^Li(p, n)^7^Be reaction is necessary. However, this can lead to a significant thermal loading. Previous researches indicated that the degradation of the Li target, including thinning and damage, was induced due to ion collisions, elevated operating temperatures, and other effects occurring from proton bombardment, and that could then reduce the neutron flux per unit of proton current^20–22,24^. Thus, the neutron flux model could represent the neutron yield reflecting each Li target condition (i.e., each treatment) by using the total number of protons as a variable. Therefore, the neutron flux model may help correct the neutron flux during treatment to deliver the prescribed dose to a patient in an AB-BNCT system. This study investigated a novel compensable approach for delivering the required neutron fluence in an AB-BNCT system employing a solid-state Li target. The suitability of the method for clinical BNCT was also evaluated.

## Methods

This study was performed at NCCH using an AB-BNCT system (Cancer Intelligence Care Systems, Inc., Tokyo, Japan (CICS)), which employs a solid-state Li target^20–22^. The system consisted of an accelerator for protons, a transport device, a target structure (containing a Li target), and a beam-shaping assembly. The ^7^Li(p,n)^7^Be reaction generated neutrons. A nominal proton current of 12 mA was delivered to the target structure, and its nominal energy was 2.5 MeV^22^. A previous study reported that the saturated radioactivity of gold can serve as a substitute for the total neutron flux, even when the Li target degradation occurs in the AB-BNCT system, which was properly designed for BNCT^21^. Although it is important for the AB-BNCT system that the epithermal neutron flux is measured^25,26^, the energy spectrum of the generated neutrons is determined by the proton energy, the proton path length in the Li target at each residual proton energy value, and the relevant aspects of the Li(p, n) reaction, such as the Q-value. In the previous study, the saturated radioactivity of gold encapsulated with and without the cadmium capsule (0.5-mm thickness) was also measured, and the cadmium ratio, which was defined as the ratio of those saturated radioactivity, was then acquired^21^. The cadmium ratio was consistent over the lifetime of Li target, and it was 1.21 ± 0.02^21^. Thus, the epithermal neutrons significantly contributed to the saturated radioactivity of gold. In addition, although the energy spectrum changed slightly due to the degradation of the Li target, a previous study reported that the total neutron flux could be evaluated by the saturated radioactivity of gold^21^. Furthermore, another study investigated the relationship between the numbers of generated neutrons and protons delivered to the target structure, suggesting that the neutron flux could be controlled by the proton current as the saturated radioactivity of gold was evaluated in Bq/mA/atom^22^. This unit can eliminate the effects of the fluctuations in the delivered protons, individual differences in each gold wire, and radioactive decay on each measurement^12,14^. Therefore, in AB-BNCT systems for clinical use, neutron beam control is performed with a cumulative sum of real-time measurements of proton charges (i.e., the required number of protons delivered to the target material)^15,16^. Furthermore, two independent ammeters in an AB-BNCT system were used to measure the proton current in real-time. Detailed descriptions of the AB-BNCT system were reported in previous studies^20–22^.

According to our previous reports, the neutron flux model can represent the expected reduction in the neutron flux per unit of proton current due to the Li target degradation. This model was established by neutron flux measurements (i.e., saturated radioactivity) and the total number of protons delivered to the Li target^22^. Additionally, the degradation did not affect the absorbed dose induced when the required neutron fluence was delivered in the BNCT^21^. Therefore, even in AB-BNCT systems employing solid-state Li targets, the neutron beam can be controlled by the number of protons delivered to the target structure when the neutron flux reduction during irradiation is corrected using the neutron flux model.

### Proposed neutron beam compensatory approach

**Figure 1:**
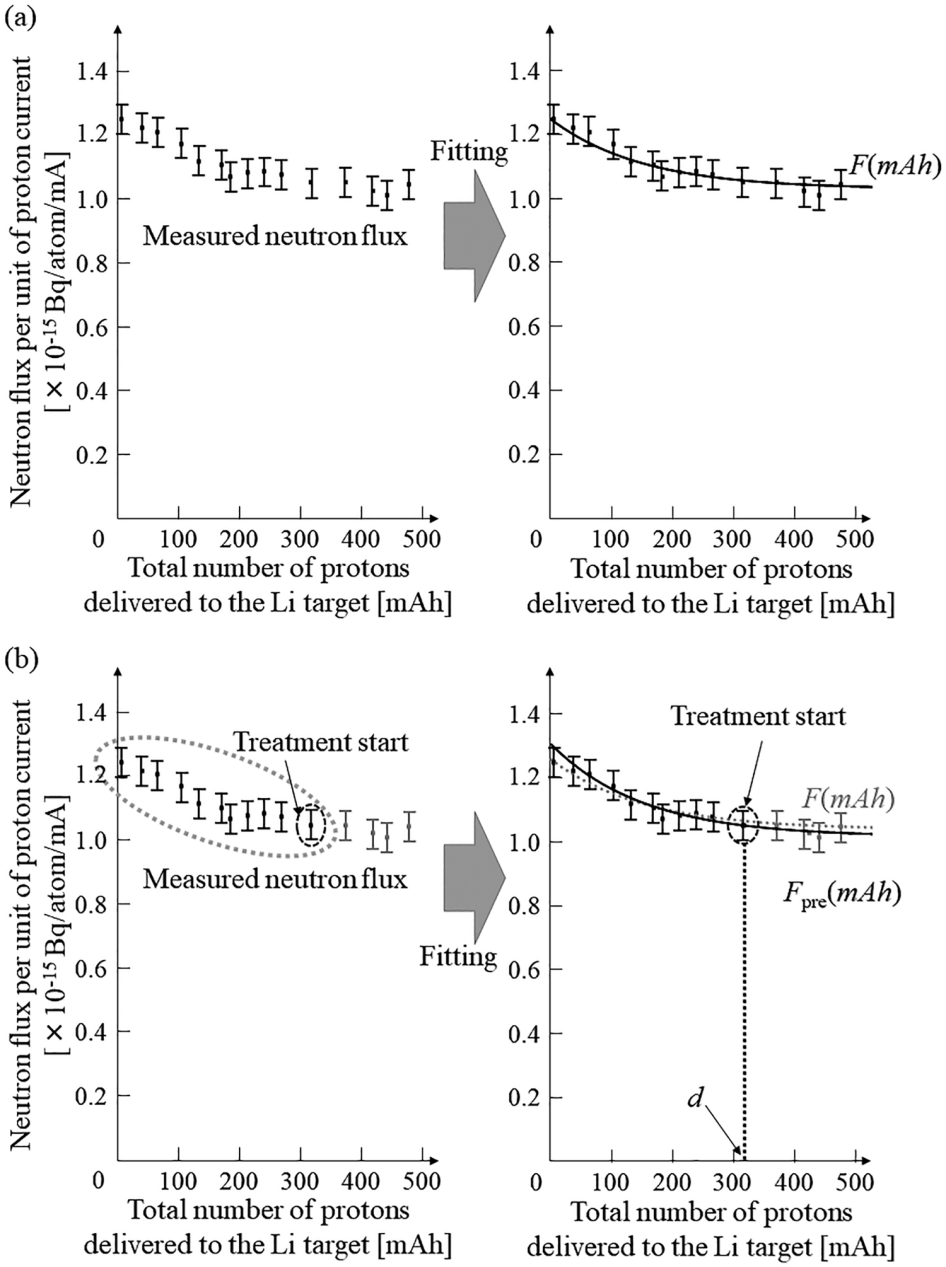
(**a**) Schematic of the neutron flux model established using all the neutron fluxes measured over the lifetime of the Li target and (**b**) the tentative neutron flux model established using the neutron fluxes measured before each treatment.

In an AB-BNCT system, neutron fluence can be calculated by integrating the neutron flux with the number of protons delivered to the target material. This is also applicable to the neutron flux model (*F*(*mAh*)) investigated in a previous study^22^, which can be expressed as follows:

1 $$F\left(mAh\right)=a\times {\text{exp}}\left(-b\times mAh\right)+c[{{\text{mA}}}^{-1}]$$

where *F* represents the neutron flux per unit of proton current based on the total number of protons delivered to the Li target; *mAh* represents the total number of protons delivered to the Li target; and *a*, *b*, and *c* are coefficients that reflect the conditions of the Li target and the reaction between the Li target and delivered protons^22^. When the neutron flux model is applied to clinical conditions, a tentative model must be established before each treatment to determine the coefficients. However, a previous study indicated that these coefficients cannot be uniquely determined. Thus, a neutron flux model was established using the measured neutron flux over the lifetime of the Li target. Figure 1 shows the neutron flux and tentative neutron flux models.

**Figure 2:**
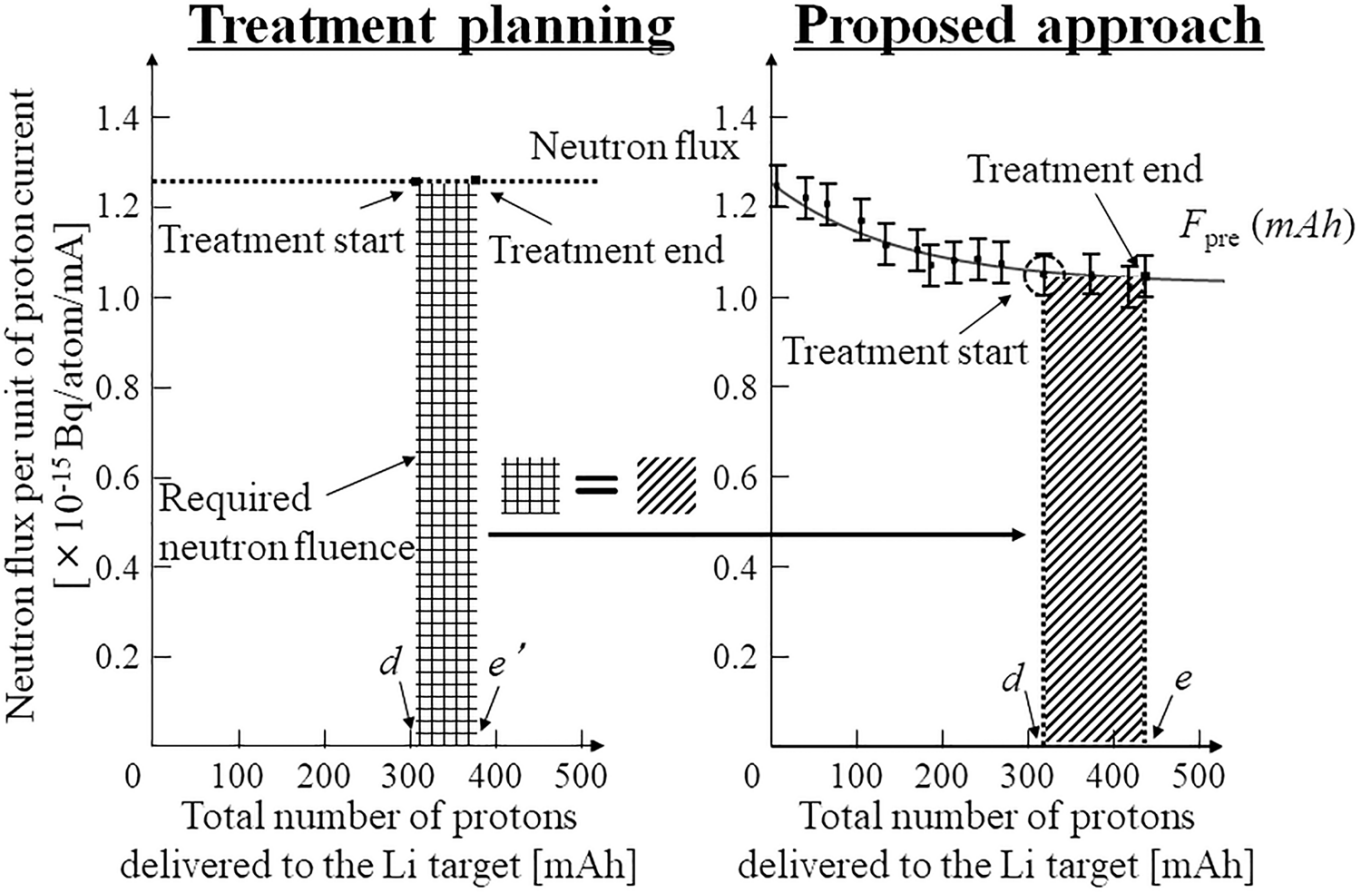
Schematic of the proposed compensatory approach for delivering the required neutron fluence.

As shown in Fig. 1, the neutron flux and tentative neutron flux models differ^22^. Additionally, the tentative neutron flux model variations were expected for each treatment. The tentative model is denoted as *F*_pre_ and is shown in Fig. 1b. The proposed approach uses the tentative model to deliver the required neutron fluence calculated during treatment planning. Figure 2 shows a schematic of the proposed compensatory approach for delivering the required neutron fluence in an AB-BNCT system.

As shown in Fig. 2, the required neutron fluence must be derived to calculate the required proton charge. Hence, the neutron flux reduction must be predicted using *F*_pre_ before treatment to calculate the proton charges required for delivering the required neutron fluence to the AB-BNCT system. This is because the neutron flux depends on each treatment (i.e., the total number of protons delivered to the Li target for each treatment), and a reduction in the neutron flux is expected during treatment. The left side of Fig. 2 shows how the required neutron fluence during treatment planning was determined. Tentative proton charges were derived from a certain neutron flux delivered in the AB-BNCT system. Thus, the required neutron fluence is equal to the neutron flux integrated by the tentative proton charges, which correspond to the integration region on the left side of Fig. 2. In the proposed approach, the required neutron fluence must be delivered considering the neutron flux reduction. The right-hand side of Fig. 2 shows a schematic of the proposed approach for delivering the required neutron fluence. The required proton charges are calculated using the tentative neutron flux model (*F*_pre_) to consider the neutron flux reduction in each treatment. The required neutron fluence is then calculated by integrating a tentative neutron flux model (*F*_pre_) with the required proton charge as follows:

2 $$NF={\int }_{d}^{e}{F}_{{\text{pre}}}\left(mAh\right)$$

where *NF* denotes the required neutron fluence, which integrates the neutron flux before and at the end of the treatment; *F*_pre_(*mAh*) denotes the tentative neutron flux model as a function of the total number of protons delivered to the Li target (i.e., *mAh*); and *d* and *e* denote the total number of protons delivered to the Li target before and after treatment, respectively. To deliver the required neutron fluence, the required proton charges (“*e*–*d*” in Fig. 2) calculated within the integration region during treatment planning (left side of Fig. 2) must be the same as that in the proposed approach (right side of Fig. 2). Therefore, the AB-BNCT system employing a Li target can deliver the required neutron fluence using the proposed approach, despite the neutron flux reduction.

### Validation of the proposed compensatory approach under clinical conditions

Because the *F*_pre_ values vary for each treatment, the proposed approach requires determining the accuracy of the required proton charges under clinical conditions. The proposed approach was validated using previously reported measured neutron flux and neutron flux models^22^. Assuming treatments at each total number of protons delivered to the Li target, the proposed approach was validated at each measurement point of the neutron flux. In each measurement interval, the accumulated proton charges delivered to the Li target were below 86.4 × 10^3^ mC, and more than 55 measurements were performed in each target. The time spent on setup, neutron irradiation, activation measurement of gold using the HP-Ge detector, and analysis was 5, 5, 10, and 5 min, respectively, and the total time reached 25 min per measurement. Five Li targets were used for validation^22^. Furthermore, a unique lot number was assigned to each target to indicate that they were not manufactured simultaneously.

Previous clinical reports using AB-BNCT systems employed irradiation times of approximately 60 min^15,16^. Another previous report indicated that using a proton current of 10–20 mA requires 1 h to deliver the required neutron fluence for BNCT with an AB-BNCT system employing a lithium target^24^. Hence, during treatment planning, this study assumed a constant neutron flux of 1.20 × 10^–15^ Bq/mA/atom and a tentative proton charge of 43.2 × 10^3^ mC (*e′*–*d* = 43.2 × 10^3^ mC; i.e., 12 mA × 1 h irradiation) to calculate the required neutron fluence. Consequently, the required neutron fluence (defined as the required integration region) was calculated as 5.18 × 10^–11^ atom^−1^. Therefore, the proposed approach can calculate the required proton charge as the required integration region for each treatment.

To validate the proposed approach, the required proton charge derived from the tentative neutron flux model for each treatment was applied to the neutron flux model. The neutron flux model was established using the measured neutron flux over the lifetime of the Li target to obtain the actual integration region, which was compared with the 5.18 × 10^–11^ atom^−1^ region.

Furthermore, to examine the usefulness of the proposed approach, the required proton charges were derived by assuming a constant neutron flux during treatment (i.e., disregarding the neutron flux reduction during treatment). The neutron flux measured immediately before treatment was regarded as its constant neutron flux, and the required proton charge was calculated as that of the required integration region (5.18 × 10^–11^ atom^−1^) in each treatment. To obtain the actual integration region, the required proton charge was applied to the neutron flux model established using the measured neutron flux over the lifetime of the Li target. This region was compared with the 5.18 × 10^–11^ atom^−1^ region.

### Statistical analysis

In the validation process, statistical analyses were performed to evaluate the discrepancies between the required and actual integration regions. The Shapiro–Wilk test was performed to determine whether the discrepancies followed a normal distribution. A paired t-test was used as a parametric test, and the Wilcoxon signed-rank test was used as a non-parametric test for comparison. The discrepancies between the five Li targets were compared, and Bartlett’s test was used to examine whether they followed a normal distribution. A one-way analysis of variance (ANOVA) and a Kruskal–Wallis test were applied to those results as a parametric and non-parametric, respectively. A *P*-value of less than 0.05 was considered statistically significant.

## Results

**Figure 3:**
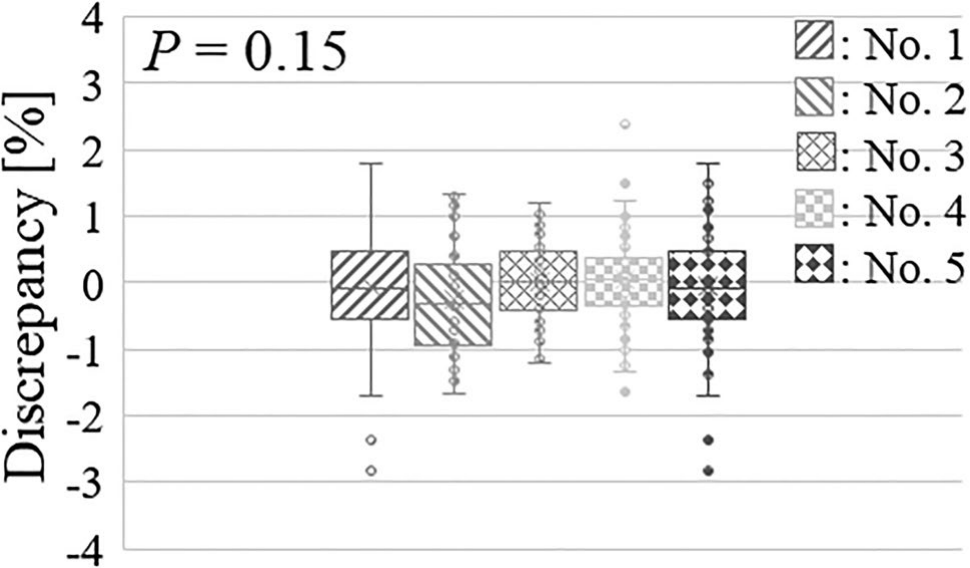
Accuracy variations in delivering the required neutron fluence using the proposed compensatory approach. The required neutron fluence was 5.18 × 10^–11^ atom^−1^ for each comparison.

The median value of the neutron flux reduction during the treatment reached 0.7% (range: 0.6–0.8%) for the five Li targets, assuming that the irradiation time was 60 min. Figure 3 shows the discrepancies between the required and actual integrated regions over the lifetimes of the five Li targets.

Based on the previous comparison, the discrepancies in Li target nos. 1–4 followed a normal distribution (*p* = 0.06, 0.12, 0.31, and 0.21, respectively, Shapiro–Wilk test), whereas those in Li target no. 5 did not (*p* = 0.04, Shapiro–Wilk test). The median discrepancies (range) for each of the five Li targets were − 0.1% (− 2.8–1.8%), − 0.3% (− 1.7–1.3%), 0.0% (− 1.2–1.2%), 0.1% (− 1.6–2.4%), and 0.1% (− 1.5–1.1%), and the absolute value of discrepancy did not exceed 3.0% in any Li target. The discrepancy rates near 1% for the five Li targets were 75.4%, 66.7%, 88.9%, 82.1%, and 94.5%. Furthermore, the discrepancies between the five Li targets did not follow a normal distribution (*p* < 0.01, Bartlett’s test) and were not statistically different (*p* = 0.15, Kruskal–Wallis test).

**Figure 4:**
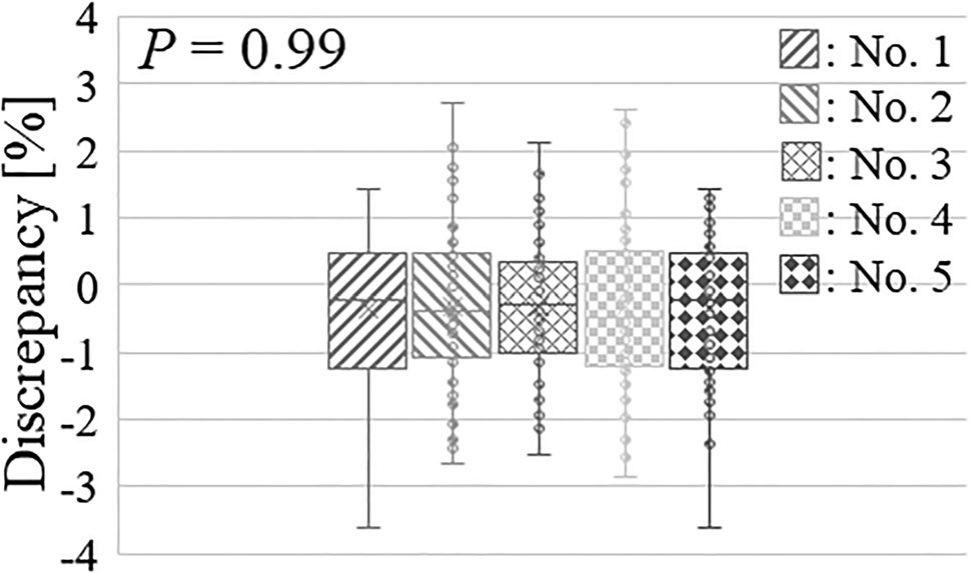
Delivering accuracy variations of the required neutron fluence using the constant neutron flux measured immediately before treatment. The required neutron fluence was 5.18 × 10^–11^ atom^−1^ for each comparison.

Figure 4 shows the discrepancies between the required and actual integrated regions over the lifetimes of the five Li targets when the required regions were calculated using the constant neutron flux measured immediately before treatment.

The comparison between the actual and required integration regions for each treatment shows that the discrepancies in each Li target followed a normal distribution (*p* = 0.17, 0.74, 0.95, 0.94, and 0.32, Shapiro–Wilk test). The mean discrepancies (range) for each of the five Li targets were − 0.4% (− 3.6–1.4%), − 0.3% (− 2.7–2.7%), − 0.3% (− 2.5–2.1%), − 0.3% (− 2.9–2.6%), and − 0.2% (− 2.2–2.4%), and a discrepancy of more than 3.0% was also observed. The standard deviations (SDs) for the five Li targets were 1.0%, 1.2%, 1.0%, 1.2%, and 1.1%. Furthermore, the discrepancies between the five Li targets did not follow a normal distribution (*p* = 0.59, Bartlett’s test). The discrepancies among the five Li targets were not statistically different (*p* = 0.99, ANOVA).

**Figure 5:**
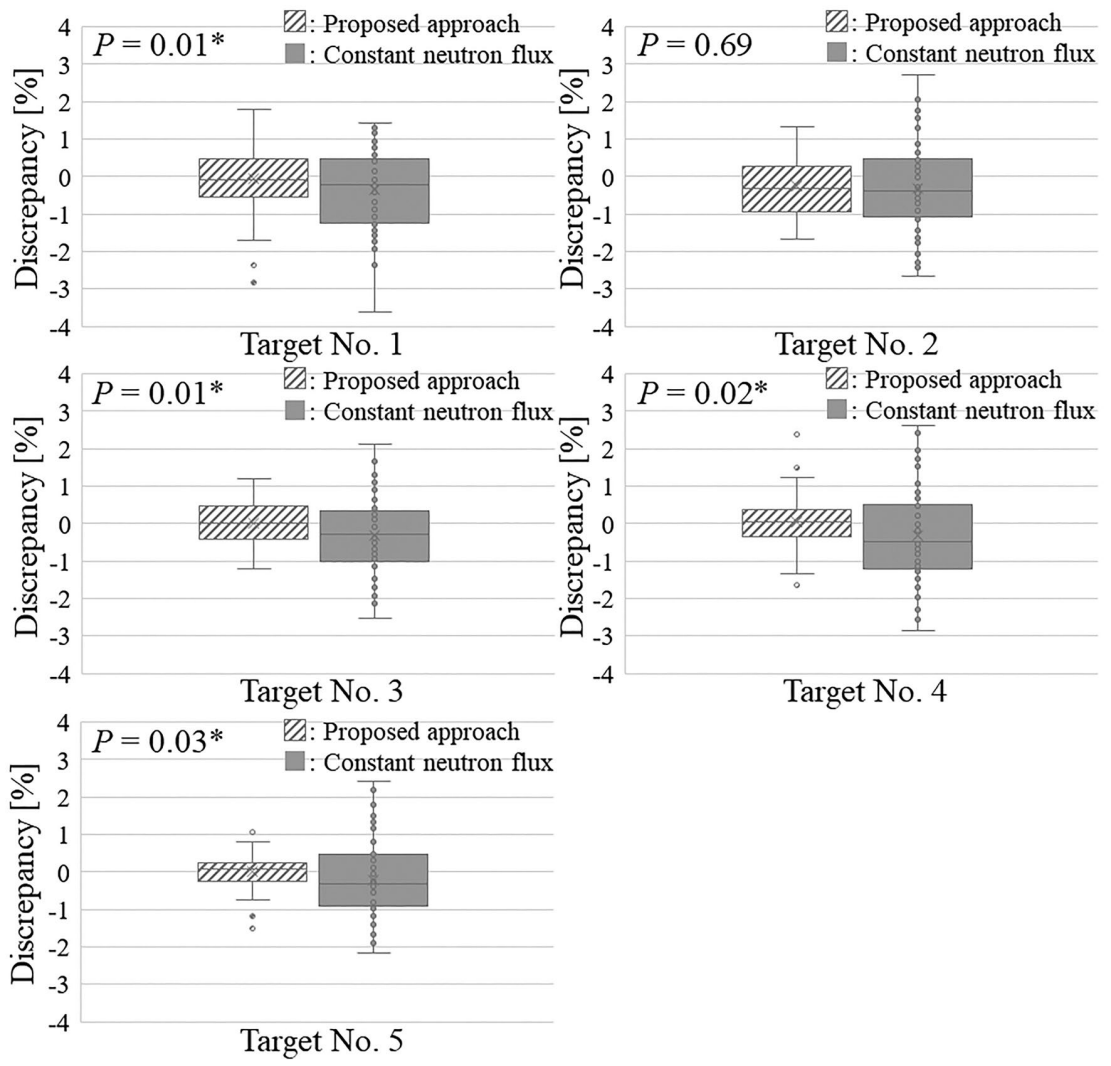
Delivering accuracies of the required neutron fluence using the proposed compensatory approach and the constant neutron flux measured immediately before the treatment in each Li target.

Figure 5 shows the discrepancies between the required and actual integration regions over the lifetime of each Li target.

According to Fig. 5, the delivering accuracy for the required neutron fluence between using the proposed approach and the constant neutron flux measured before treatment shows statistically significant differences in Li targets no. 1, 3, 4, and 5 (*p* = 0.01, 0.01, 0.02, and 0.03, respectively) and did not show for the Li target no. 2 (*p* = 0.69). Note that the comparison in Li targets no. 1–4 was performed using the paired-t test, and that in no. 5 was performed using the Wilcoxon signed-rank test. These results indicate that the discrepancies between the prescribed dose and the actual delivered dose were within 3.0% using the proposed compensatory approach while those exceeded 3.0% without it.

## Discussion

This study investigates a novel compensatory approach for delivering the required neutron fluence to a patient in an AB-BNCT system employing a solid-state Li target. The system considers the Li target degradation because several protons must be delivered to the target to obtain the required number of neutrons for BNCT^20^. According to previous reports, the degradation is associated with a neutron flux reduction over the lifetime of the Li target, which can be expressed as a function of the total number of protons delivered to the Li target^22^. However, when the required neutron fluence for BNCT was delivered, no notable effect on the absorbed dose was observed, even if degradation occurred during treatment^21^. Thus, this study proposes a novel compensatory approach for delivering the required neutron fluence to a patient by considering the neutron flux reduction utilizing the neutron flux model. The proposed approach considers the reduction in neutron flux during treatment and over the lifetime of the Li target. As shown in Fig. 5, the proposed approach can improve the accuracy of delivering the required neutron fluence to a patient. Therefore, this study investigated the usefulness of the proposed approach for an AB-BNCT system employing a Li target.

A previous study indicated that the neutron flux model (utilized in the proposed approach) was established using all the neutron fluxes measured over the lifetime of the Li target, which enabled the evaluation of the actual neutron flux at each total number of protons delivered to the Li target^22^. Furthermore, each Li target must be established^22^. However, a tentative neutron flux model must be established for each treatment to apply the neutron flux model to the proposed approach. As shown in Fig. 3, the required number of neutrons delivered to a patient was determined over the lifetime of the Li target using the proposed approach. In particular, the required neutron fluence could be delivered within 3% and was independent of the target. In contrast, the accuracy of delivering the required neutron fluences exceeded 3% in some cases when the neutron flux reduction was disregarded. Furthermore, a previous study reported that the fission chamber was used for the real-time neutron monitor in the BNCT system^27^. According to the previous report, a difference of more than 5% was present between the measured neutrons in the phantom placed on the patient position and those measured using the real-time monitor^27^. Thus, the proposed method can more accurately deliver the required neutron fluences.

Conversely, because the neutron flux reduction was near 1% during treatment, the required neutron fluence could be delivered using the neutron flux measured immediately before treatment without considering the reduction. In this case, as shown in Fig. 4, larger variations in the accuracy of delivering the required neutron fluences are expected. A reason for this is the propagation of uncertainty in the neutron flux measurement contributing to the accuracy of delivering the required neutron fluence. As shown in Fig. 4, the variations in the accuracy of delivering the required neutron fluence for each Li target are larger than those of the proposed approach. The SDs for each Li target ranged from 1.0 to 1.2%. According to a previous report, these results were consistent with the discrepancies between the measured and calculated neutron fluxes derived from the neutron flux model for each total number of protons delivered to the Li target. Furthermore, the maximum discrepancy between the five Li targets was 3.6%. This is because the discrepancies reflected the uncertainty of the neutron flux measurement (2.6%) and the neutron flux reduction during treatment^21^. The proposed compensatory approach required the relative measurement data of the neutron flux reduction based on the total number of protons delivered to the Li target rather than the absolute measurement. The previous reports indicated that the measurement uncertainty of the thermal neutron fluences ranged from 5 to 7%, where this measurement uncertainty included the uncertainty of the HP-Ge detector efficiency^28^. Furthermore, another report indicated that the measurement uncertainty of the radioactivity of gold was 1.5%, and it might exclude the uncertainty of the HP-Ge detector efficiency^29^. The uncertainty in this study did not include the HP-Ge detector efficiency. Instead, the placements of the gold for the neutron irradiation and the measurement on the HP-Ge detector, the weight of each gold, and the number of delivered protons were included. This was because the measurement geometry was consistent for each measurement using the HP-Ge detector. Therefore, the measurement uncertainty of 2.6% was comparable to the previous report.

This study had the following limitations. The proton current was restricted to 12.0 mA, and the cooling efficiency of the Li target was neglected. A previous study reported that the neutron flux reduction depended on the thermal load on the Li target, and the larger thermal load on the Li target induced a greater reduction^20^. Thus, the neutron flux during treatment might increase when applying a proton current higher than 12.0 mA. However, as the proposed approach accounts for the neutron flux reduction during treatment, it becomes crucial when applying higher proton currents. Furthermore, in AB-BNCT systems, future developments may increase the proton current to increase the neutron flux. Even if higher proton currents are applied, the proposed approach could be applied as long as the neutron flux over the lifetime of the Li target is measured with the assumed proton current. This is because the coefficients of the neutron flux model used in the proposed approach account for the Li target condition (i.e., the thermal load on the Li target)^22^. Thus, we expect to implement the proposed control method under various conditions and report its results.

## Conclusions

This study proposed a novel compensatory approach for delivering the required neutron fluence to a patient in an AB-BNCT system employing a solid-state Li target. The proposed approach considered neutron flux reduction and decreased the effect of uncertainty in neutron flux measurements when delivering the required neutron fluence. Therefore, this study revealed that the proposed approach could improve the accuracy of delivering the required neutron fluence for BNCT, even if a neutron flux reduction is expected during treatment and over the lifetime of the Li target. Furthermore, research and developments regarding AB-BNCT systems have been active recently, and further development is expected. One is using a higher proton current to increase the neutron flux. Even in that case, the proposed approach may be applied for delivering the required neutrons by selecting the adequate coefficient of the neutron flux model used in the proposed approach, and our approach becomes crucial, even though the accuracy of delivering the required neutron fluence will be discussed in the future work. Therefore, the proposed control method applies be applicable to other BNCT systems employing the Li target, contributing to the further development of the AB-BNCT system. We expect to implement the proposed method in non-clinical studies and clinical practice to improve therapeutic efficacy and safety of the AB-BNCT.

## Data Availability

Data is provided within the manuscript.
